# Tailoring biopsy strategy in the MRI-fusion prostate biopsy era: systematic, targeted or neither?

**DOI:** 10.1186/s12894-024-01553-1

**Published:** 2024-08-07

**Authors:** Fredrik Jäderling, Martin Bergman, Jan Chandra Engel, Ashkan Mortezavi, Wolfgang Picker, Erik Skaaheim Haug, Martin Eklund, Tobias Nordström

**Affiliations:** 1 Department of Medical Epidemiology and Biostatistics, Karolinska Institutet, Stockholm, S-171 77 Sweden; 2 Department of Molecular Medicine and Surgery, Karolinska Institutet, Stockholm, Sweden; 3 Department of Clinical Sciences at Danderyd Hospital, Karolinska Institutet, Stockholm, Sweden; 4 Department of Radiology, Capio S:T Görans Hospital, Stockholm, Sweden; 5 Department of Surgery, Capio S:T Görans Hospital, Stockholm, Sweden; 6 Department of Urology, University Hospital Zurich, Zurich, Switzerland; 7 Department of Urology, Karolinska University Hospital Solna, Stockholm, Sweden; 8 Department of Radiology, Aleris Cancer Center, Oslo, Norway; 9 Section of Urology, Vestfold Hospital Trust, Tønsberg, Norway

**Keywords:** Prostate cancer, Prostate neoplasm, Magnetic resonance imaging, MRI, Prostate biopsies

## Abstract

**Background:**

Magnetic  resonance imaging (MRI) followed by targeted biopsy (TBx) is utilized for prostate cancer (PCa) detection. However, the value of adding systematic biopsies (SBx) to targeted biopsy procedures (combined biopsy; CBx) in men with suspicious MRI findings has not been determined.

**Methods:**

We analysed biopsy outcomes in 429 men with MRI lesions in the prospective multicenter STHLM3MRI pilot study, planned for prostate biopsy. Participants underwent 1.5T biparametric MRI without contrast enhancement, reported according to the PI-RADS v2, and with TBx plus SBx if the MRI lesion score was ≥ 3. The endpoints were clinically nonsignificant (nsPCa) and clinically significant PCa (csPCa), defined as ISUP grade groups 1 and ≥ 2, respectively.

**Results:**

The median age was 65 years (59–70), and the median PSA 6.0 ng/ml (4.1–9.0). The detection rates of csPCa when using TBx or SBx combined were 18%, 46%, and 85% in men with PIRADS scores of 3 (*n* = 195), 4 (*n* = 121), and 5 (*n* = 113), respectively. This combined strategy detected csPCa in more men than TBx alone (43.6% vs 39.2%, *p* < 0.02), with similar detection of nsPCa (19.3% vs 17.7%, *p* = 0.2).

In men with equivocal lesions (PI-RADS 3), the detection rates for csPCa were similar for the combined strategy and for TBx alone (17.9% and 15.4%, *p* = 0.06). However, there was an increase in the detection of nsPCa when using the combined strategy (21.0% vs 15.4%, *p* < 0.02).

Men with equivocal lesions and a PSA density < 0.1 ng/ml^2^ or a Stockholm 3 test < 0.11 had a low risk of harboring csPCa.

**Conclusions:**

Supplementing targeted with systematic biopsies enhances clinically significant cancer detection. However, in men with equivocal lesions, this combination has potential for detecting nonsignificant disease. A subgroup of men with equivocal MRI findings may be identified as having a low risk for significant cancer and spared unnecessary biopsies.

**Supplementary Information:**

The online version contains supplementary material available at 10.1186/s12894-024-01553-1.

## Introduction

The most common form of cancer in men is detected via tissue sampling, and approximately 1,000,000 prostate biopsy procedures are performed annually in Europe. Multiparametric magnetic resonance imaging (mpMRI) has been shown to accurately identify lesions harboring significant cancer [1]. Subsequently, several studies have shown improved cancer detection using MRI and fusion targeted biopsies in clinical-practice cohorts [2–5] as well as in a screening setting [6, 7]. Thus, international guidelines recommend performing multiparametric magnetic resonance imaging (MRI) before prostate biopsy decisions are made [8, 9].

For men with MRI lesions suspicious of prostate cancer, a number of strategies might be applied to maximize the detection of clinically significant prostate cancer and simultaneously limit the risk of overdetection, harm to patients and resource overuse in the health care system. Such strategies might include adding systematic biopsies to the target biopsy procedure or omitting biopsies completely in subsets of men with a low risk of cancer.

Few studies have reported the value of adding systematic biopsies to an MRI-targeted biopsy pathway, specifically in men with significant lesions, representing the cohort of men in whom targeted biopsies can be performed [2, 3, 10]. In particular, in light of the high number of utilized biopsy techniques and MRI protocols, there is a need to further illustrate the impact of systematic biopsies on cancer detection when applied in target biopsy settings.

Using data from the prospective STHLM3MRI pilot study [11], we report (1) the value of systematic to targeted biopsies for the detection of prostate cancer in clinical practice and (2) exploring patient groups with equivocal lesions where prostate biopsy might be omitted.

## Subjects and methods

### Study design and participants

The STHLM3MRI pilot study was a prospective, multicentre, paired diagnostic trial [11]. Participants were recruited from three Scandinavian sites between May 2016 and June 2017. Men aged 45–75 years were eligible for inclusion if they had no prior diagnosis of PCa, with a PSA ≥ 3 ng/ml and were referred for a PCa workup (Supplementary Table 1). The STHLM3MRI pilot study was performed before the main STHLM3MRI study (not reported here) [12].

All men underwent a prostate MRI 15-min biparametric protocol on a 1.5-T MRI without contrast enhancement (see Supplementary Table 2 for protocol). Study participants were instructed to refrain from sexual activity 3 days before the MRI examination. To optimise image quality a minimal enema (Microlax) was administered a few hours prior to the examination, and just before the examination intramuscular glucagon (1 mg) or Buscopan was given. MRI scans were reported according to the PI-RADS v2 "assessment without adequate DCE” by one dedicated prostate MRI radiologist at each of the three study sites; up to three lesions assessed as PI-RADS score ≥ 3 were marked. Reported lesions were segmented in a dedicated software followed by two to three targeted biopsies taken using software MRI-TRUS fusion biopsy (Koelis (Oslo), Artemis (Tönsberg), BioJet (Stockholm)), and thereafter a 12-core systematic biopsy taken from the dorsal prostate (four biopsies from right to left in the base, mid and apex of the prostate) [11]. For this analysis, we included only men who underwent biopsy for at least one significant lesion, defined as a PI-RADS score ≥ 3, on MRI.

The primary definition of csPCa was an ISUP grade ≥ 2 (Gleason ≥ 7) according to either systematic or targeted biopsy. An alternative definition of csPCa was ISUP GG ≥ 3. Nonsignificant prostate cancer (nsPCa) was defined as a Gleason score of 6/ISUP GG1. For detailed descriptions of the study design and population, see Grönberg et al. [11] and Nordström et al. [13]. The regional ethics committees of Stockholm and Oslo approved the study (Swedish ethical review authority Dnr. 2016/392–31 and Regional Comittees for Medical Research Ethics South East Norway Dnr. 2016/684). Prior to inclusion, patients provided written informed consent to participate in the study.

We report observed biopsy outcomes stratified by the maximum PI-RADS score. The results were further stratified by previously suggested cut-offs for PSA (10 ng/ml), prostate volume (50 cc), PSA density (0.15 ng/ml^2^) and the Stockholm 3 risk score (15% risk of ISUP GG ≥ 2 cancer) [12, 14, 15]. Proportions were compared using the McNemar test, where *p* < 0.05 was considered to indicate statistical significance. All analyses were performed using Stata/MP 13.1 (Stata Corp., College Station, TX).

## Results

A total of 429 (81%) men out of 532 men who underwent MRI had at least one significant lesion (PI-RADS ≥ 3) and underwent combined biopsy procedure including targeted and systematic biopsies. The median age and PSA level in this cohort were 65 years (interquartile range (IQR)) and 6 ng/ml (IQR 4.0–8.0), respectively. A quarter of the participants (25%, *n* = 107) had a previous biopsy (Table 1).

**Table 1:** Patient characteristics of men with visible MRI lesions in the STHLM3MRI pilot study

	**All significant lesions**	**Equivocal lesions**
PI-RADS class (n)	3–5 (*n* = 429)	3 (*n* = 195)
Age, years (median, IQR)	56 (50–63)	58 (51–63)
PSA, ng/ml (median, IQR)	6.0 (4.1–9.0)	6.0 (4.0–8.0)
Previous biopsy (n, %)	107 (25.0%)	55 (28.2%)
Digital rectal examination		
T1	275 (66.0%)	162 (85.7%)
T2	118 (28.3%)	26 (13.8%)
T3	22 (5.3%)	1 (0.5%)
T4	2 (0.5%)	0
Missing	12	6
PI-RADS score		
3	195 (45.5%)	195 (100%)
4	121 (28.2%)	
5	113 (26.3%)	
Number of MRI lesions		
1	250 (58.3%)	129 (66.2%)
2	129 (30.1%)	52 (26.7%)
3	50 (11.7%)	14 (7.2%)

Participants in the STHLM3MRI pilot study (*n* = 532) who had lesions on MRI (*n* = 429). All men were clinical patients planned for prostate biopsy or pre-biopsy MRI; All men underwent MRI + target biopsy + systematic biopsy

*PI-RADS* Prostate Imaging Reporting and Data System, *IQR* Interquartile range, *PSA* Prostate-specific antigen, *MRI* Magnetic resonance imaging

### Cancer detection in men with significant MRI lesions

**Fig. 1:**
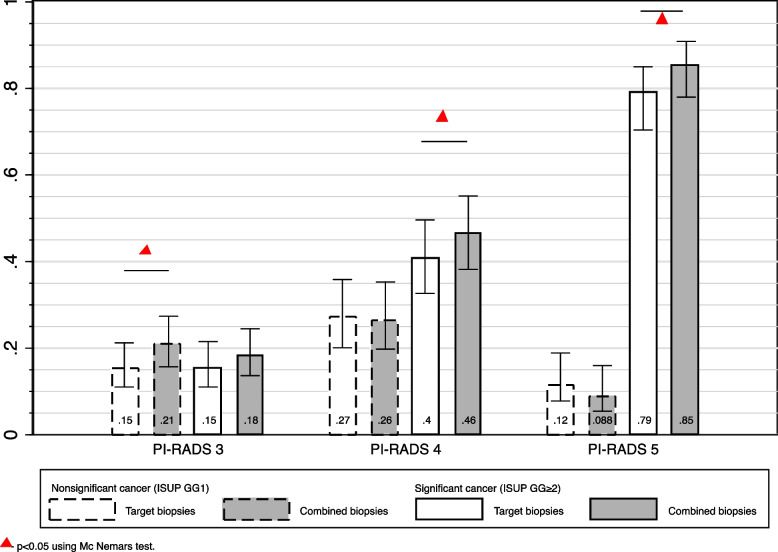
Proportion of cancer findings in 429 men according to lesion characterization on MRI (PI-RADS v2)

The incidence of csPCa after combined biopsy was 35/195 (17.9%; 95% CI: 13.2–24.0) in men with PI-RADS 3 lesions, 56/121 (46.3%; 95% CI: 37.5–55.3) in those with PI-RADS 4 lesions, and 96/113 (85.0%; 95% CI: 77.1–90.5) in those with PI-RADS 5 lesions (Fig. 1 and Table 2). 10% (19/189) men with csPCa had their tumor detected only by systematic biopsy (14 ISUP GG2, 1 GG3 and 3 GG ≥ 4) Supplementary Tables 3 and 5 tabulates all the biopsy findings in this study. Figure 1 shows the relative detection of significant and insignificant cancers by targeted and combined biopsy procedures via MRI. In men with suspicious MRI lesions (PI-RADS ≥ 3), the combined biopsy procedure vs targeted biopsy alone detected 43.6% (95% CI 38.9–48.3) vs 39.2% (95% CI 34.6–43.9) of clinically significant cancers, suggesting that the value of systematic biopsy is 4.4% (Table 2). This difference in detection was also statistically significant for an alternative definition of clinically significant cancer (ISUP ≥ 3 grade group: 20.7% vs. 18.4%; *p* < 0.02).

**Table 2:** Detection of cancer in 429 men with at least one significant lesion on MRI

		All suspicious lesions (PI-RADS 3–5) 429 patients			Equivocal lesions (PI-RADS 3) 195 patients		
		ISUP GG 1	ISUP GG ≥ 2	ISUP GG ≥ 3	ISUP GG 1	ISUP GG ≥ 2	ISUP GG ≥ 3
Systematic biopsy	N % (95% CI)	87 20.3% (16.7–24.4)	156 36.4% (31.9–41.0)	67 15.6% (12.5–19.4)	41 21.0% (15.8–27.4)	25 12.8% (8.8–18.4)	7 3.6% (1.7–7.4)
Targeted biopsy	N % (95% CI)	76 17.7% (14.4–21.6)	168 39.2% (34.6–43.9)	79 18.4% (15.0–22.4)	30 15.4% (10.9–21.2)	30 15.4% (10.9–21.2)	11 5.6% (3.1–9.9)
Systematic and targeted biopsy	N % (95% CI)	83 19.3% (15.9–23.4)	187 43.6% (38.9–48.3)	89 20.7% (17.2–24.9)	41 21.0% (15.8–27.4)	35 17.9% (13.1–24.0)	12 6.2% (3.5–10.6)
Added detection of systematic biopsy		1.6%	4.4%	2.3%	5.6%	2.5%	0.5%
*p*-value (TBx vs TBx + Sbx)		0.23	** < 0.02**	** < 0.02**	** < 0.02**	0.06	1

### Cancer detection in men with equivocal MRI lesions

In men with equivocal lesions on MRI (PI-RADS 3), the detection of nonsignificant cancer was greater if a combined biopsy procedure was used compared to a targeted-only strategy (21.0% vs 15.4%; difference 5.6%; *p* < 0.02); however, there was no statistically significant difference in the detection of csPCa. (detection difference 2.5%, *p* = 0.06).

### Identification of men at low risk of finding clinically significant cancer on biopsy

Based on a priori decided and previously suggested cut-offs, we assessed the risk of significant cancer in men stratified according to PSA level, PSA density or Stockholm 3 risk score. Among men with any significant MRI lesion (PIRADS score 3–5), none of the cut-offs used, identified a group of men with < 11% risk of ISUP GG ≥ 2 cancer (resembling the risk among men with PSA ≤ 3 ng/ml in a screening population [7, 16, 17]). However, the presuggested cut-off values were associated with delaying the detection of significant cancer. Therefore, exploratory cut-offs for men with equivocal lesions (PI-RADS 3) are illustrated in Table 3. We found that a PSA density < 0.1 ng/ml^2^ is a reasonable alternative for identifying men with a PI-RADS 3 at low risk of cancer while saving one-third of biopsy procedures in these men and delaying diagnosis for 11% of patients with ISUP GG ≥ 2 cancer. Similarly, men with a Stockholm 3 < 0.11 and a PI-RADS 3 had a 5% risk of ISUP GG ≥ 2 cancer. Excluding biopsy procedures in these men would spare 52% of biopsy procedures, decreasing ISUP 1 detection by 46% and delaying diagnosis for 14% of the ISUP ≥ 2 cancers otherwise detected (Table 3). Alternative cut-offs for men with a PI-RADS 3 lesion, either a PSA density < 0.15 ng/ml^2^, a prostate volume ≥ 50 cc or a Stockholm 3 score < 15%, could be used for identifying men with a low risk of clinically significant cancer via combined biopsy (10.3%, 7.2%, and 6.8%, respectively). Omitting biopsy in these men saved 16–19% (69–83/429) of the biopsy procedures in this study (Supplementary Table 4).

**Table 3:** Risk of finding significant cancer (ISUP ≥ 2) in 195 men with PI-RADS 3 findings on MRI according to the PSA level, PSA density and Stockholm 3 (S3). Stipulated cut-offs in bold

		**Findings in men with equivocal lesions (PI-RADS 3)**				
		**Performed biopsies**	**Saved biopsies**	**Delayed detection of ISUP 1**	**Delayed detection of ISUP 2**	**Risk of ISUP ≥ 2 in performed bx**
		N (%)	N (%)	N (%)	N (%)	% (95% CI)
Total		195 (100)	195 (100)	40 (100)	35 (100)	17.9% (13.1–24.0)
PSA	< 4	162 (83)	33 (17)	8 (20)	3 (9)	9.1% (2.9–25.2)
	< 6	103 (52)	92 (48)	20 (50)	16 (46)	17.4% (10.9–26.7)
	< 8	56 (27)	139 (73)	26 (73)	26 (74)	17.3% (9.2–30.3)
PSA density	< 0.05	181 (92.5)	14 (7.5)	3 (7.5)	0 (0)	0
	< 0.07	160 (82)	35 (18)	7 (18)	1 (3)	2.9% (0.3–18.3)
	** < 0.10**	**133 (67)**	**62 (33)**	**14 (35)**	**4 (11)**	**6.5% (2.4–16.2)**
	< 0.12	108 (54)	87 (46)	17 (43)	8 (23)	9.2% (4.6–17.5)
	< 0.15	76 (37)	119 (63)	27 (68)	13 (37)	10.9% (6.4–18.0)
S3 score	< 7	126 (65)	69 (35)	13 (32)	1(3)	1.4 (0.1–9.8)
	** < 11**	**94 (48)**	**101 (52)**	**19 (46)**	**5 (14)**	**5.0 (2.1–11.4)**
	< 15	78 (40)	117 (60)	26 (63)	8 (22)	6.8 (3.4–13.2)
	< 17	67 (44)	128 (66)	27 (66)	10 (28)	7.8 (4.2–14.0)

### Contralateral cancer findings

In men with unilateral (left-sided or right-sided) lesions on MRI, 25.6% and 27.2%, respectively, had contralateral cancer findings on systematic biopsy. The available data did not allow for Gleason grading by biopsy core (Table 4).

**Table 4:** Proportion of 429 men with significant lesions on MRI showing any contralateral cancer findings on systematic biopsies by lesion location

	**Systematic biopsy findings**				
	Left		Right		Total
**Significant lesion on MRI (PIRADS ≥ 3)**	Cancer	Benign	Cancer	Benign	
Left	44% (72)	56% (90)	27% (44)	73% (118)	162
Right	26% (34)	74% (99)	44% (59)	56% (74)	133
Bilateral	49% (60)	51% (63)	49% (60)	51% (63)	123
Missing	(4)	(7)	(3)	(8)	11
Total	170	259	166	263	429

## Discussion

### Overall findings

Using data from the prospective, paired-design STHLM3MRI pilot study, we report biopsy outcomes in men with significant lesions on MRI defined as any lesion scoring PI-RADS ≥ 3. We found that adding systematic biopsies to a targeted biopsy procedure increases the detection of clinically significant cancer. Furthermore, we found no statistically significant difference in the detection of clinically significant cancer when systematic biopsies were added to targeted biopsies in men with equivocal MRI lesions (PI-RADS 3); instead, there was an increased risk of overdiagnosis of insignificant cancers when systematic biopsies were added. Finally, we report that a subset of men with equivocal lesions might be identified as having a low risk of clinically significant cancer and thus be saved from a biopsy procedure based on their prostate volume, prostate-specific antigen (PSA) density or Stockholm 3 risk score.

Several other studies have compared detection in targeted and systematic biopsies, frequently also reporting on total cancer detection using the combined modality.

Rouviere et al. report on 251 men undergoing systematic and targeted biopsies using a paired design where 198 had a significant Likert-scored lesion [3]. Since 5.2% of significant cancers were missed by targeted biopsies, they suggest that systematic biopsies should not be omitted in men undergoing prostate MRI. Although not explicitly reported, one can, however, note that approximately 40% of the added effect results from systematic biopsies performed in the subset of *men without MRI lesions*. In the MRI-first study, combined biopsies detected more nonsignificant cancers than did targeted biopsies alone, thus increasing the risk of overdetection.

Elkhoury et al. reported a paired design in 300 men in which 248 had visible MRI lesions; cancer detection rates were approximately 60% with either systematic or fusion biopsies and 70% in total when both systematic, cognitive fusion biopsies and software fusion biopsies were performed. They report discordance of tumor locations strengthening the suggestion that the different biopsy strategies detect different tumors. Therefore, combining targeted and systematic biopsies was suggested [10]. Even though the combined strategy increased the detection of significant prostate cancer in their study, the consequences regarding overdetection are not clearly presented.

A third study by van der Leest also uses a paired design, implementing in-bore biopsies to PI-RADS ≥ 3 lesions in addition to systematic biopsies. They selectively report 7% higher detection rate of significant cancer if systematic biopsies are added to targeted in men with significant MRI lesions [2].

A review also including earlier studies indicated a nonsignificant 5 percentage point increase in significant cancer detection and a simultaneous sharp 12 percentage point increase in the detection of nonsignificant cancer; thus, a strict targeted biopsy approach was suggested [18].

Our results add to the published evidence indicating a limited 5–10 percentage points benefit in the detection of significant lesions by adding systematic biopsies to targeted lesions in men with MRI. This benefit depends on several factors, including the underlying disease prevalence and the quality of both MRI and biopsy procedures, indicating that additional studies are warranted. Furthermore, by adding systematic biopsies, a nonnegligible risk of an increase in overdiagnosis follows. We illustrate that this effect is most prominent in men with equivocal lesions where cancer detection rates are lower.

Our results support the addition of systematic biopsies to targeted biopsies in men with a higher risk of cancer findings on targeted biopsies (e.g. PI-RADS ≥ 4), giving additional staging information before treatment decisions (e.g. information on contralateral cancer) are made. Despite being out of the scope of this study, adding systematic biopsies to targeted might also limit the risk of over-assessment of disease risk associated with a targeted-only approach to prostate biopsy [21].

Our study has several strengths. First, this multicentre, prospective study used a paired design and was specifically designed to study the real-life performance of targeted biopsies. We used a structured and high-quality short radiology protocol developed for the early detection of prostate cancer and highly experienced uro-radiologists to ensure high radiological quality. However, there are also limitations. This pragmatic study performed in clinical practice included data from several clinical departments (urology/radiology/pathology/biobank/laboratory) in a complex logistic chain. MRI was executed with a bi-parametric protocol at 1.5T magnet field strength with a large proportion of PI-RADS 3 at three different sites in clinical practice with variable MRI experience between sites as described in the original study. Although the quality of the data was monitored continuously, some final data were missing. Second, the systematic biopsy were performed unblinded from the MRI results, possibly affecting the results of the systematic biopsies. Thirdly, one centre recently introduced soft-ware fusion-guided biopsies, and the learning curve for the procedure has previously been described [19]. Fourth, with the paired design of our study comes that any addition of biopsy needles in the diagnostic process (e.g. systematic biopsies) increases cancer detection. Finally, since only cancer finding, but not Gleason grading, was available in the data, conclusion from the analysis on laterality should be made with caution. The statistical significance of these findings is therefore strongly dependent on the study size, and the findings should be interpreted with caution. Finally, although it has previously been shown that TBx decreases disease misclassification [20], in the absence of prostatectomy specimens, the true disease prevalence is unknown.

## Conclusion

The addition of systematic biopsies when performing targeted biopsies in men with significant MRI lesions increases the detection of significant cancer and should thus be considered in clinical practice. Systematic biopsies might, however, be omitted when performing targeted biopsies in men with equivocal MRI lesions due to an increased risk of overdiagnosis. Men with equivocal MRI lesions and a low PSA density or low Stockholm 3 score have a low risk of clinically significant prostate cancer.

### Supplementary Information

Supplementary Material 1.

## Data Availability

Data supporting the results of the manuscript can be made available upon request to the last author; Tobias Nordström (tobias.nordstrom@ki.se) after approval.

## Abbreviations

CBx: Combined biopsy
SBx: Systematic biopsy
TBx: Targeted biopsy
nsPCa: Nonsignificant prostate cancer
csPCa: Clinically significant prostate cancer
MRI: Magnetic resonance imaging
mpMRI: Multi-parametric magnetic resonance imaging
PI-RADS: Prostate imaging-Reporting and data system
STHLM3MRI: Stockholm 3 magnetic resonance imaging
ISUP: International society of urological pathology
GG: Grade group
IQR: Inter quartile range
