# Supplementary material for: Tailoring biopsy strategy in the MRI-fusion prostate biopsy era: systematic, targeted or neither?
Source: BMC Urol. 2024 Aug 7;24:168. doi: 10.1186/s12894-024-01553-1 (PMC11304837; doi:10.1186/s12894-024-01553-1)
Supplement: Supplementary file 1 — Supplementary Material 1. [file 12894_2024_1553_MOESM1_ESM.docx]

|  | **Total Patients (n=532)** | **Participants by lesion on MRI** | |
| --- | --- | --- | --- |
|  |  | PIRADS ≥3 (n=429) | PIRADS 1-2 (n=103) |
| Age, years (median, IQR) | 58 (51-64) | 56 (50-63) | 63 (59-68) |
| PSA, ng/ml (median, IQR) | 6.3 (4.6-9.0) | 6.0 (4.1-9.0) | 7.0 (5.0-10.0) |
| Previous biopsy (n, %) | 145 (27.3%) | 107 (25.0%) | 38 (36.9%) |
|  |  |  |  |
| Digital rectal examination |  |  |  |
| T1 | 366 (71.2%) | 275 (66.0%) | 91 (93.8%) |
| T2 | 124 (24.1%) | 118 (28.3%) | 6 (6.2%) |
| T3 | 22 (4.3%) | 22 (5.3%) |  |
| T4 | 2 (0.4%) | 2 (0.5%) |  |
| Missing | 18 | 12 | 6 |
| PI-RADSv2 score |  |  |  |
| 1 | 2 (0.4%) |  | 2 (1.9%) |
| 2 | 101 (19.0%) |  | 101 (98.1%) |
| 3 | 195 (36.7%) | 195 (45.5%) |  |
| 4 | 121 (22.7%) | 121 (28.2%) |  |
| 5 | 113 (21.2%) | 113 (26.3%) |  |

Supplementary table 1: Patient characteristics in STHLM3MRI pilot study

|  | **Sequence** | | | | |
| --- | --- | --- | --- | --- | --- |
| **Variable** | **Plane-loc**  **Haste** | **T2-weighted**  **SPACE (3D)** | **T1-weighted vibe-twist**  **(Dixon)** | **DWI** | **DWI** |
| Orientation | 3-plane | axial | axial | axial | axial |
| Acquired Voxel size (mm) | 1.8 x 1.8 x 8.0 | 0.9 x 0.9 x 0.9 | 1.1 x 1.1 x 3.0 | 2.25 x 2.25 x 4.0 | 2.0 x 2.0 x 4.0 |
| Field-of-view (FoV) read, mm | 450 | 290 | 160 | 180 | 160 |
| Number of slices | 7 cor  5 sag  5 axial | 104 | 40 | 20 | 20 |
| Time to repetition (TR), ms | 700 | 1500 | 7.37 | 3000 | 3100 |
| Time to echo (TE), ms | 90 | 181 | 1. 2.39 2. 4.77 | 56 | 67 |
| b-values | - | - | - | 100, 450, 800 | 1500 |
| Acquisition time  min:sec | 0:12 | 7:29 | 0:33 | 3:53 | 3:23 |
| Comment |  | Reconstructions:  sagittal  coronal |  | Calculated  b=1500 and  ADC |  |
| DWI=Diffusion weighted imaging, ADC=Apparent diffusion coeffient, SPACE= Sampling Perfection with Application optimized Contrasts using different flip angle Evolution | | | | | |

Supplementary table 2: Magnetic resonance imaging (MRI) detection protocol used in STHLM3 MRI study prior to transrectal ultrasound/MRI-fusion guided prostate biopsies.

Total acquisition time 15 minutes and 30 seconds. Scanners used in the study 1.5T Siemens Magnetom Avanto-fit (Oslo), Aera (Stockholm) and Aera (Tønsberg).

| Target biopsy findings | Target+Systematic biopsy findings | | | | | | | |
| --- | --- | --- | --- | --- | --- | --- | --- | --- |
|  | ISUP | 0 | 1 | 2 | | 3 | ≥4 | Total |
|  | 0 | 159 | 16 | 6 | | 1 | 3 | 185 |
|  | 1 | 0 | 67 | 9 | | 0 | 0 | 76 |
|  | 2 | 0 | 0 | 83 | | 6 | 0 | 89 |
|  | 3 | 0 | 0 | 0 | | 37 | 3 | 40 |
|  | ≥4 | 0 | 0 | 0 | | 0 | 39 | 39 |
|  | Total | 159 | 83 | 98 | 44 | | 45 | 429 |

|  | PI-RADS 3 | | | | | | | |
| --- | --- | --- | --- | --- | --- | --- | --- | --- |
| Target biopsy findings | Target+Systematic biopsy findings | | | | | | | |
|  | ISUP | 0 | 1 | 2 | | 3 | ≥4 | Total |
|  | 0 | 119 | 13 | 2 | | 1 | 0 | 135 |
|  | 1 | 0 | 28 | 2 | | 0 | 0 | 30 |
|  | 2 | 0 | 0 | 19 | | 0 | 0 | 19 |
|  | 3 | 0 | 0 | 0 | | 6 | 0 | 6 |
|  | ≥4 | 0 | 0 | 0 | | 0 | 5 | 5 |
|  | Total | 119 | 41 | 23 | 7 | | 5 | 195 |

|  | PI-RADS 4 | | | | | | | |
| --- | --- | --- | --- | --- | --- | --- | --- | --- |
| Target biopsy findings | Target+Systematic biopsy findings | | | | | | | |
|  | ISUP | 0 | 1 | 2 | | 3 | ≥4 | Total |
|  | 0 | 33 | 2 | 2 | | 0 | 2 | 39 |
|  | 1 | 0 | 30 | 3 | | 0 | 0 | 33 |
|  | 2 | 0 | 0 | 28 | | 2 | 0 | 30 |
|  | 3 | 0 | 0 | 0 | | 11 | 0 | 11 |
|  | ≥4 | 0 | 0 | 0 | | 0 | 8 | 8 |
|  | Total | 33 | 32 | 33 | 13 | | 10 | 121 |

|  | PI-RADS 5 | | | | | | | |
| --- | --- | --- | --- | --- | --- | --- | --- | --- |
| Target biopsy findings | Target+Systematic biopsy findings | | | | | | | |
|  | ISUP | 0 | 1 | 2 | | 3 | ≥4 | Total |
|  | 0 | 7 | 1 | 2 | | 0 | 1 | 11 |
|  | 1 | 0 | 9 | 4 | | 0 | 0 | 13 |
|  | 2 | 0 | 0 | 36 | | 4 | 0 | 40 |
|  | 3 | 0 | 0 | 0 | | 20 | 3 | 23 |
|  | ≥4 | 0 | 0 | 0 | | 0 | 26 | 26 |
|  | Total | 7 | 10 | 42 | 24 | | 30 | 113 |

Supplementary tables 3: Cross-tabulation biopsy findings in 429 men with significant (PIRADS≥3) lesions on MRI (target vs target+systematic biopsy).

|  |  | **All significant lesions (PI-RADS 3-5)** | | | **Equivocal lesions (PI-RADS 3)** | | |
| --- | --- | --- | --- | --- | --- | --- | --- |
|  |  |  | **Risk of clinically significant cancer (ISUP GG≥2)** | |  | **Risk of clinically significant cancer (ISUP GG≥2)** | |
|  |  | **n** | **Targeted biopsies** | **Targeted+systematic biopsies** | **n** | **Targeted biopsies** | **Targeted+systematic biopsies** |
| Total |  | 429 | 39.2% (34.6–43.9) | 43.6% (38.9–48.3) | 195 | 15.4% (10.9–21.2) | 17.9% (13.1–24.0) |
| PSA | <10 | 334 | 33.5% (28.7–38.8) | 38.9% (33.8–44.2) | 161 | 14.3% (9.6–20.6) | 17.4% (12.2–24.1) |
|  | ≥10 | 95 | 58.9% (48.7–68.4) | 60.0% (49.8–69.4) | 34 | 20.6% (10.0–37.3) | 20.6% (10.0–37.7) |
| PSA density | <0.15 | 231 | 22.1% (17.2–27.9) | 27.3% (21.9–33.4) | 126 | 8.7% (4.9%–15.1%) | 10.3% (6.1–17.0) |
|  | ≥0.15 | 198 | 59.1% (52.1–65.8) | 62.6% (55.6–69.1) | 69 | 27.5% (18.2–39.4) | 31.9% (21.9–43.9) |
| Prostate volume | <50 | 283 | 47.7% (41.9–53.6) | 52.7% (46.8–58.4) | 112 | 22.3% (15.5–31.1) | 25.9% (18.6–34.9) |
|  | ≥50 | 146 | 22.6% (16.5–30.1) | 26.0% (19.5–33.8) | 83 | 6.0% (2.5–13.8) | 7.2% (3.3–15.3) |
| Stockholm3 score | <15 | 172 | 11.0% (7.1–16.7) | 14.0% (9.5–20.0) | 117 | 6.0% (2.9–12.1) | 6.8% (3.4–13.2) |
|  | ≥15 | 257 | 58.0% (51.2–63.9) | 63.4% (57.3–69.1) | 78 | 29.5% (20.3–40.6) | 34.6% (24.8–45.9) |

Supplementary table 4: Detection of significant cancer (ISUP GG ≥2) in 429 men with at least one suspicious lesion on MRI by subgroups

|  |  | | | | | | | |
| --- | --- | --- | --- | --- | --- | --- | --- | --- |
| Target biopsy findings | Target+Systematic biopsy findings | | | | | | | |
|  | ISUP | 0 | 1 | 2 | | 3 | ≥4 | Total |
|  | 0 | 157 | 14 | 7 | | 4 | 4 | 186 |
|  | 1 | 15 | 54 | 12 | | 2 | 4 | 87 |
|  | 2 | 5 | 9 | 64 | | 9 | 2 | 89 |
|  | 3 | 1 | 0 | 6 | | 22 | 5 | 34 |
|  | ≥4 | 3 | 0 | 0 | | 3 | 27 | 33 |
|  | Total | 181 | 77 | 89 | 40 | | 42 | 429 |

Supplementary Table 5: List of cancerfindings by biopsy type in 429 men with at least one suspicious lesion on MRI
